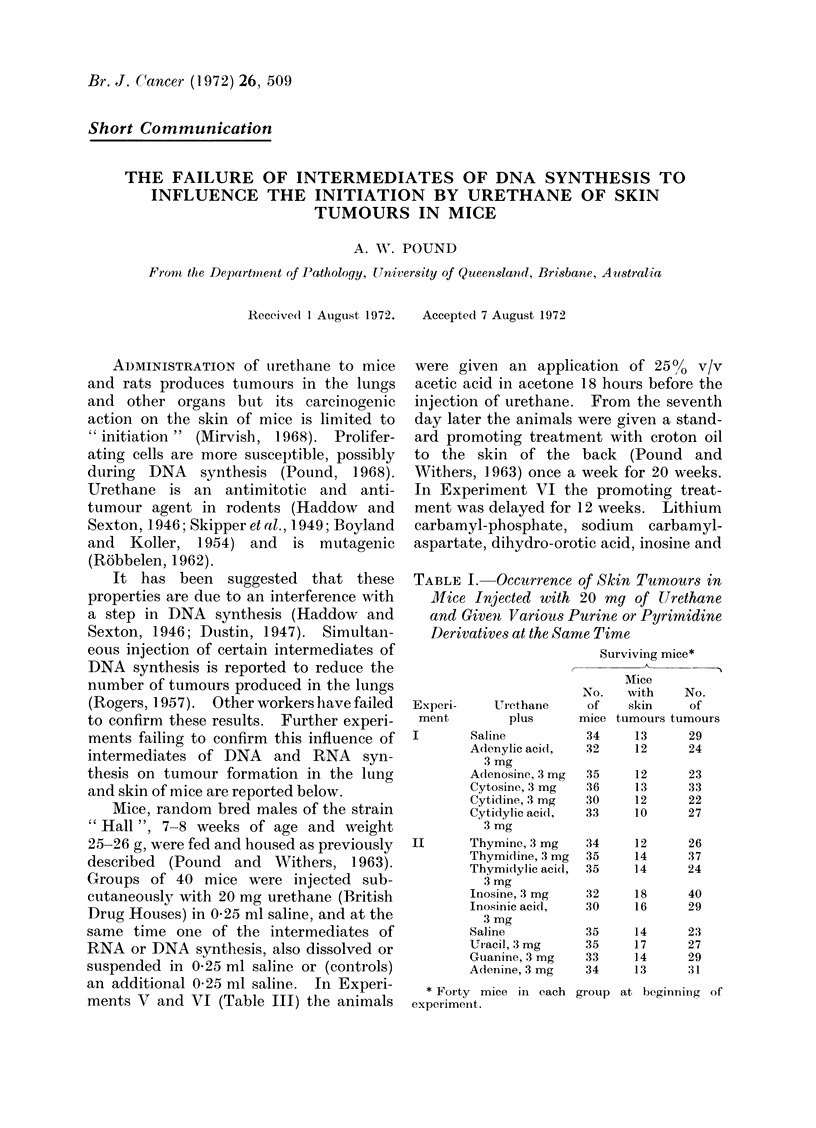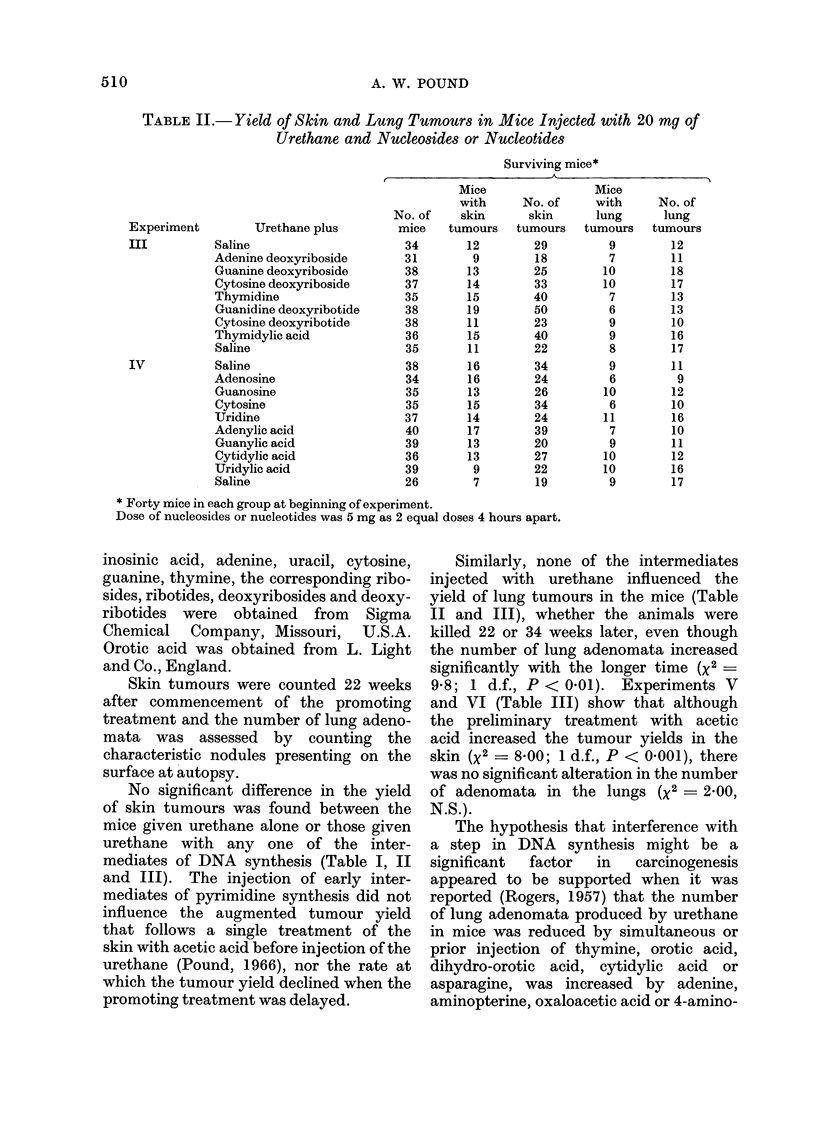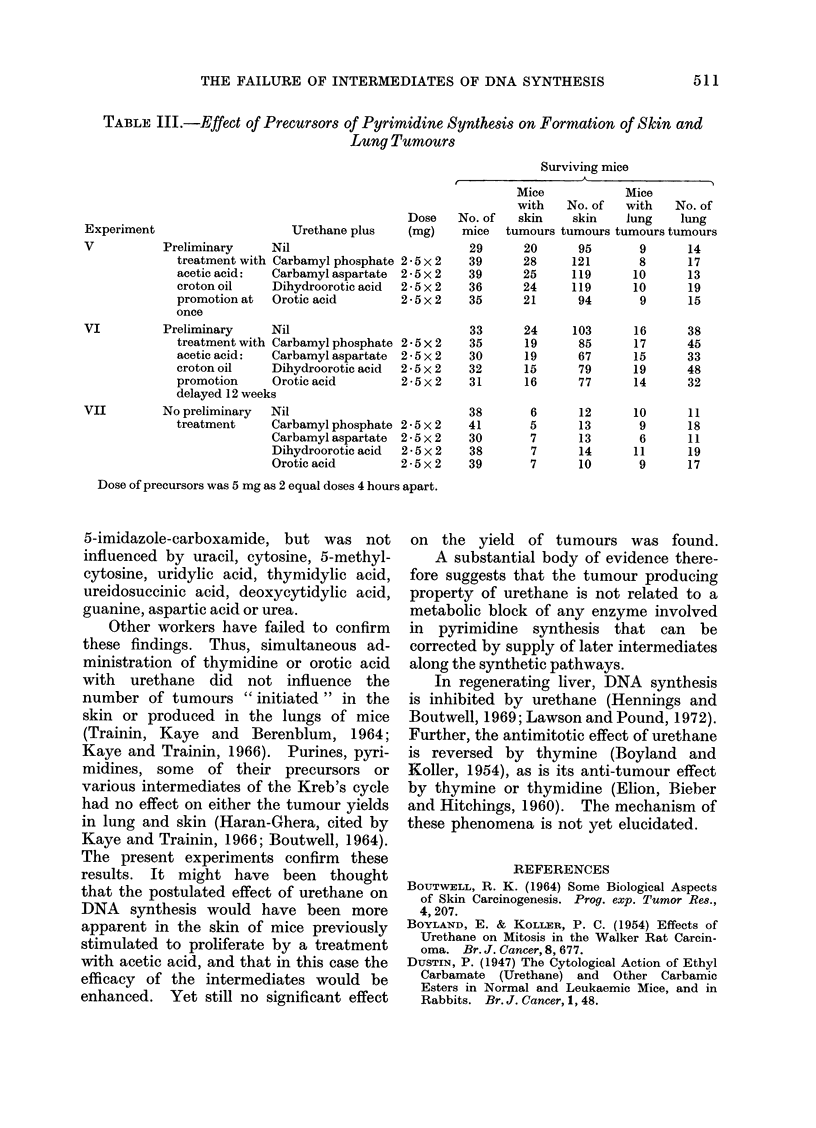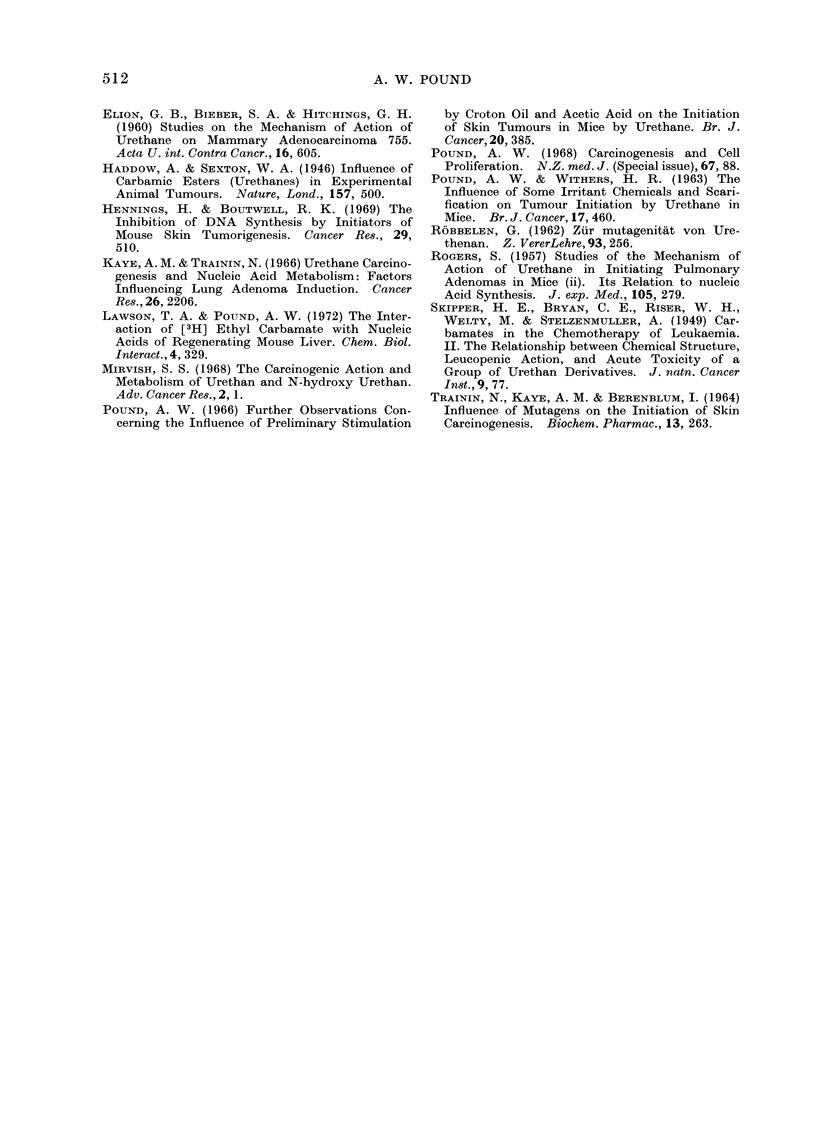# The failure of intermediates of DNA synthesis to influence the initiation by urethane of skin tumours in mice.

**DOI:** 10.1038/bjc.1972.70

**Published:** 1972-12

**Authors:** A. W. Pound


Br. J. Cancer (1972) 26, 509

*Short Communication*

### THE FAILURE OF INTERMEDIATES OF DNA SYNTHESIS TO INFLUENCE THE INITIATION BY URETHANE OF SKIN TUMOURS IN MICE

A. W. POUND

*From the Department of Pathology, University of Queensland, Brisbane, Australia*

Received 1 August 1972. Accepted 7 August 1972

ADMINISTRATION of urethane to mice and rats produces tumours in the lungs and other organs but its carcinogenic action on the skin of mice is limited to "initiation" (Mirvish, 1968). Proliferating cells are more susceptible, possibly during DNA synthesis (Pound, 1968). Urethane is an antimitotic and antitumour agent in rodents (Haddow and Sexton, 1946; Skipper *et al.*, 1949; Boyland and Koller, 1954) and is mutagenic (Röbbelen, 1962).

It has been suggested that these properties are due to an interference with a step in DNA synthesis (Haddow and Sexton, 1946; Dustin, 1947). Simultaneous injection of certain intermediates of DNA synthesis is reported to reduce the number of tumours produced in the lungs (Rogers, 1957). Other workers have failed to confirm these results. Further experiments failing to confirm this influence of intermediates of DNA and RNA synthesis on tumour formation in the lung and skin of mice are reported below.

Mice, random bred males of the strain "Hall", 7–8 weeks of age and weight 25–26 g, were fed and housed as previously described (Pound and Withers, 1963). Groups of 40 mice were injected subcutaneously with 20 mg urethane (British Drug Houses) in 0·25 ml saline, and at the same time one of the intermediates of RNA or DNA synthesis, also dissolved or suspended in 0·25 ml saline or (controls) an additional 0·25 ml saline. In Experiments V and VI (Table III) the animals were given an application of 25% v/v acetic acid in acetone 18 hours before the injection of urethane. From the seventh day later the animals were given a standard promoting treatment with croton oil to the skin of the back (Pound and Withers, 1963) once a week for 20 weeks. In Experiment VI the promoting treatment was delayed for 12 weeks. Lithium carbamyl-phosphate, sodium carbamyl-aspartate, dihydro-orotic acid, inosine and

TABLE I.—*Occurrence of Skin Tumours in Mice Injected with 20 mg of Urethane and Given Various Purine or Pyrimidine Derivatives at the Same Time*

| | | Surviving mice* | | |
|---|---|---|---|---|
| Experiment | Urethane plus | No. of mice | Mice with skin tumours | No. of tumours |
| I | Saline | 34 | 13 | 29 |
| | Adenylic acid, 3 mg | 32 | 12 | 24 |
| | Adenosine, 3 mg | 35 | 12 | 23 |
| | Cytosine, 3 mg | 36 | 13 | 33 |
| | Cytidine, 3 mg | 30 | 12 | 22 |
| | Cytidylic acid, 3 mg | 33 | 10 | 27 |
| II | Thymine, 3 mg | 34 | 12 | 26 |
| | Thymidine, 3 mg | 35 | 14 | 37 |
| | Thymidylic acid, 3 mg | 35 | 14 | 24 |
| | Inosine, 3 mg | 32 | 18 | 40 |
| | Inosinic acid, 3 mg | 30 | 16 | 29 |
| | Saline | 35 | 14 | 23 |
| | Uracil, 3 mg | 35 | 17 | 27 |
| | Guanine, 3 mg | 33 | 14 | 29 |
| | Adenine, 3 mg | 34 | 13 | 31 |

* Forty mice in each group at beginning of experiment.

TABLE II.—*Yield of Skin and Lung Tumours in Mice Injected with* 20 *mg of Urethane and Nucleosides or Nucleotides*

| Experiment | Urethane plus | Surviving mice* No. of mice | Mice with skin tumours | No. of skin tumours | Mice with lung tumours | No. of lung tumours |
|---|---|---|---|---|---|---|
| III | Saline | 34 | 12 | 29 | 9 | 12 |
| | Adenine deoxyriboside | 31 | 9 | 18 | 7 | 11 |
| | Guanine deoxyriboside | 38 | 13 | 25 | 10 | 18 |
| | Cytosine deoxyriboside | 37 | 14 | 33 | 10 | 17 |
| | Thymidine | 35 | 15 | 40 | 7 | 13 |
| | Guanidine deoxyribotide | 38 | 19 | 50 | 6 | 13 |
| | Cytosine deoxyribotide | 38 | 11 | 23 | 9 | 10 |
| | Thymidylic acid | 36 | 15 | 40 | 9 | 16 |
| | Saline | 35 | 11 | 22 | 8 | 17 |
| IV | Saline | 38 | 16 | 34 | 9 | 11 |
| | Adenosine | 34 | 16 | 24 | 6 | 9 |
| | Guanosine | 35 | 13 | 26 | 10 | 12 |
| | Cytosine | 35 | 15 | 34 | 6 | 10 |
| | Uridine | 37 | 14 | 24 | 11 | 16 |
| | Adenylic acid | 40 | 17 | 39 | 7 | 10 |
| | Guanylic acid | 39 | 13 | 20 | 9 | 11 |
| | Cytidylic acid | 36 | 13 | 27 | 10 | 12 |
| | Uridylic acid | 39 | 9 | 22 | 10 | 16 |
| | Saline | 26 | 7 | 19 | 9 | 17 |

* Forty mice in each group at beginning of experiment.
Dose of nucleosides or nucleotides was 5 mg as 2 equal doses 4 hours apart.

inosinic acid, adenine, uracil, cytosine, guanine, thymine, the corresponding ribosides, ribotides, deoxyribosides and deoxyribotides were obtained from Sigma Chemical Company, Missouri, U.S.A. Orotic acid was obtained from L. Light and Co., England.

Skin tumours were counted 22 weeks after commencement of the promoting treatment and the number of lung adenomata was assessed by counting the characteristic nodules presenting on the surface at autopsy.

No significant difference in the yield of skin tumours was found between the mice given urethane alone or those given urethane with any one of the intermediates of DNA synthesis (Table I, II and III). The injection of early intermediates of pyrimidine synthesis did not influence the augmented tumour yield that follows a single treatment of the skin with acetic acid before injection of the urethane (Pound, 1966), nor the rate at which the tumour yield declined when the promoting treatment was delayed.

Similarly, none of the intermediates injected with urethane influenced the yield of lung tumours in the mice (Table II and III), whether the animals were killed 22 or 34 weeks later, even though the number of lung adenomata increased significantly with the longer time ($\chi^2 = 9{\cdot}8$; 1 d.f., $P < 0{\cdot}01$). Experiments V and VI (Table III) show that although the preliminary treatment with acetic acid increased the tumour yields in the skin ($\chi^2 = 8{\cdot}00$; 1 d.f., $P < 0{\cdot}001$), there was no significant alteration in the number of adenomata in the lungs ($\chi^2 = 2{\cdot}00$, N.S.).

The hypothesis that interference with a step in DNA synthesis might be a significant factor in carcinogenesis appeared to be supported when it was reported (Rogers, 1957) that the number of lung adenomata produced by urethane in mice was reduced by simultaneous or prior injection of thymine, orotic acid, dihydro-orotic acid, cytidylic acid or asparagine, was increased by adenine, aminopterine, oxaloacetic acid or 4-amino-

TABLE III.—*Effect of Precursors of Pyrimidine Synthesis on Formation of Skin and Lung Tumours*

| Experiment | | Urethane plus | Dose (mg) | Surviving mice: No. of mice | Mice with skin tumours | No. of skin tumours | Mice with lung tumours | No. of lung tumours |
|---|---|---|---|---|---|---|---|---|
| V | Preliminary | Nil | | 29 | 20 | 95 | 9 | 14 |
| | treatment with | Carbamyl phosphate | 2·5 × 2 | 39 | 28 | 121 | 8 | 17 |
| | acetic acid: | Carbamyl aspartate | 2·5 × 2 | 39 | 25 | 119 | 10 | 13 |
| | croton oil | Dihydroorotic acid | 2·5 × 2 | 36 | 24 | 119 | 10 | 19 |
| | promotion at once | Orotic acid | 2·5 × 2 | 35 | 21 | 94 | 9 | 15 |
| VI | Preliminary | Nil | | 33 | 24 | 103 | 16 | 38 |
| | treatment with | Carbamyl phosphate | 2·5 × 2 | 35 | 19 | 85 | 17 | 45 |
| | acetic acid: | Carbamyl aspartate | 2·5 × 2 | 30 | 19 | 67 | 15 | 33 |
| | croton oil | Dihydroorotic acid | 2·5 × 2 | 32 | 15 | 79 | 19 | 48 |
| | promotion delayed 12 weeks | Orotic acid | 2·5 × 2 | 31 | 16 | 77 | 14 | 32 |
| VII | No preliminary | Nil | | 38 | 6 | 12 | 10 | 11 |
| | treatment | Carbamyl phosphate | 2·5 × 2 | 41 | 5 | 13 | 9 | 18 |
| | | Carbamyl aspartate | 2·5 × 2 | 30 | 7 | 13 | 6 | 11 |
| | | Dihydroorotic acid | 2·5 × 2 | 38 | 7 | 14 | 11 | 19 |
| | | Orotic acid | 2·5 × 2 | 39 | 7 | 10 | 9 | 17 |

Dose of precursors was 5 mg as 2 equal doses 4 hours apart.

5-imidazole-carboxamide, but was not influenced by uracil, cytosine, 5-methylcytosine, uridylic acid, thymidylic acid, ureidosuccinic acid, deoxycytidylic acid, guanine, aspartic acid or urea.

Other workers have failed to confirm these findings. Thus, simultaneous administration of thymidine or orotic acid with urethane did not influence the number of tumours "initiated" in the skin or produced in the lungs of mice (Trainin, Kaye and Berenblum, 1964; Kaye and Trainin, 1966). Purines, pyrimidines, some of their precursors or various intermediates of the Kreb's cycle had no effect on either the tumour yields in lung and skin (Haran-Ghera, cited by Kaye and Trainin, 1966; Boutwell, 1964). The present experiments confirm these results. It might have been thought that the postulated effect of urethane on DNA synthesis would have been more apparent in the skin of mice previously stimulated to proliferate by a treatment with acetic acid, and that in this case the efficacy of the intermediates would be enhanced. Yet still no significant effect on the yield of tumours was found.

A substantial body of evidence therefore suggests that the tumour producing property of urethane is not related to a metabolic block of any enzyme involved in pyrimidine synthesis that can be corrected by supply of later intermediates along the synthetic pathways.

In regenerating liver, DNA synthesis is inhibited by urethane (Hennings and Boutwell, 1969; Lawson and Pound, 1972). Further, the antimitotic effect of urethane is reversed by thymine (Boyland and Koller, 1954), as is its anti-tumour effect by thymine or thymidine (Elion, Bieber and Hitchings, 1960). The mechanism of these phenomena is not yet elucidated.

### REFERENCES

BOUTWELL, R. K. (1964) Some Biological Aspects of Skin Carcinogenesis. *Prog. exp. Tumor Res.*, **4**, 207.

BOYLAND, E. & KOLLER, P. C. (1954) Effects of Urethane on Mitosis in the Walker Rat Carcinoma. *Br. J. Cancer*, **8**, 677.

DUSTIN, P. (1947) The Cytological Action of Ethyl Carbamate (Urethane) and Other Carbamic Esters in Normal and Leukaemic Mice, and in Rabbits. *Br. J. Cancer*, **1**, 48.

ELION, G. B., BIEBER, S. A. & HITCHINGS, G. H. (1960) Studies on the Mechanism of Action of Urethane on Mammary Adenocarcinoma 755. *Acta U. int. Contra Cancr.*, **16**, 605.

HADDOW, A. & SEXTON, W. A. (1946) Influence of Carbamic Esters (Urethanes) in Experimental Animal Tumours. *Nature, Lond.*, **157**, 500.

HENNINGS, H. & BOUTWELL, R. K. (1969) The Inhibition of DNA Synthesis by Initiators of Mouse Skin Tumorigenesis. *Cancer Res.*, **29**, 510.

KAYE, A. M. & TRAININ, N. (1966) Urethane Carcinogenesis and Nucleic Acid Metabolism: Factors Influencing Lung Adenoma Induction. *Cancer Res.*, **26**, 2206.

LAWSON, T. A. & POUND, A. W. (1972) The Interaction of [$^3$H] Ethyl Carbamate with Nucleic Acids of Regenerating Mouse Liver. *Chem. Biol. Interact.*, **4**, 329.

MIRVISH, S. S. (1968) The Carcinogenic Action and Metabolism of Urethan and N-hydroxy Urethan. *Adv. Cancer Res.*, **2**, 1.

POUND, A. W. (1966) Further Observations Concerning the Influence of Preliminary Stimulation by Croton Oil and Acetic Acid on the Initiation of Skin Tumours in Mice by Urethane. *Br. J. Cancer*, **20**, 385.

POUND, A. W. (1968) Carcinogenesis and Cell Proliferation. *N.Z. med. J.* (Special issue), **67**, 88.

POUND, A. W. & WITHERS, H. R. (1963) The Influence of Some Irritant Chemicals and Scarification on Tumour Initiation by Urethane in Mice. *Br. J. Cancer*, **17**, 460.

RÖBBELEN, G. (1962) Zür mutagenität von Urethenan. *Z. VererLehre*, **93**, 256.

ROGERS, S. (1957) Studies of the Mechanism of Action of Urethane in Initiating Pulmonary Adenomas in Mice (ii). Its Relation to nucleic Acid Synthesis. *J. exp. Med.*, **105**, 279.

SKIPPER, H. E., BRYAN, C. E., RISER, W. H., WELTY, M. & STELZENMULLER, A. (1949) Carbamates in the Chemotherapy of Leukaemia. II. The Relationship between Chemical Structure, Leucopenic Action, and Acute Toxicity of a Group of Urethan Derivatives. *J. natn. Cancer Inst.*, **9**, 77.

TRAININ, N., KAYE, A. M. & BERENBLUM, I. (1964) Influence of Mutagens on the Initiation of Skin Carcinogenesis. *Biochem. Pharmac.*, **13**, 263.